# Enhancement of Peroxidase Stability Against Oxidative Self-Inactivation by Co-immobilization with a Redox-Active Protein in Mesoporous Silicon and Silica Microparticles

**DOI:** 10.1186/s11671-016-1605-4

**Published:** 2016-09-20

**Authors:** P. Sahare, M. Ayala, R. Vazquez-Duhalt, U. Pal, A. Loni, L. T. Canham, I. Osorio, V. Agarwal

**Affiliations:** 1 Centro de Investigacion en Ingenieria y Ciencias Aplicadas, Universidad Autónoma del Estado de México, Av. Univ. 1001, Col. Chamilpa, Cuernavaca, Morelos 62209 Mexico; 2 Instituto de Biotecnología, Universidad Nacional Autónoma de México, Av. Universidad 2001, Chamilpa, Cuernavaca, 62210 Morelos Mexico; 3 Centro de Nanociencias y Nanotecnología, Universidad Nacional Autónoma de Mexico, Km. 107, Carretera Tijuana-Ensenada, Apdo. Postal 14, CP 22800 Ensenada, Baja California Mexico; 4 Instituto de Física, Benemérita Universidad Autónoma de Puebla, Puebla, Mexico; 5 pSiMedica Ltd, Malvern Hills Science Park, Geraldine Road, Malvern, Worcestershire WR14 3SZ UK; 6 Facultad de Química, Pontificia Universidad Católica de Chile, Santiago, Chile

**Keywords:** Porous silica, Porous silicon, Microparticles, Peroxidase, Auto-inactivation

## Abstract

The study of the stability enhancement of a peroxidase immobilized onto mesoporous silicon/silica microparticles is presented. Peroxidases tend to get inactivated in the presence of hydrogen peroxide, their essential co-substrate, following an auto-inactivation mechanism. In order to minimize this inactivation, a second protein was co-immobilized to act as an electron acceptor and thus increase the stability against self-oxidation of peroxidase. Two heme proteins were immobilized into the microparticles: a fungal commercial peroxidase and cytochrome c from equine heart. Two types of biocatalysts were prepared: one with only covalently immobilized peroxidase (one-protein system) and another based on covalent co-immobilization of peroxidase and cytochrome c (two-protein system), both immobilized by using carbodiimide chemistry. The amount of immobilized protein was estimated spectrophotometrically, and the characterization of the biocatalyst support matrix was performed using Brunauer–Emmett–Teller (BET), scanning electron microscopy with energy-dispersive X-ray spectroscopy (SEM-EDX), and Fourier transform infrared (FTIR) analyses. Stability studies show that co-immobilization with the two-protein system enhances the oxidative stability of peroxidase almost four times with respect to the one-protein system. Thermal stability analysis shows that the immobilization of peroxidase in derivatized porous silicon microparticles does not protect the protein from thermal denaturation, whereas biogenic silica microparticles confer significant thermal stabilization.

## Background

Enzymes are proven to be very efficient catalysts for biochemical reactions. Industrially important enzymes require higher productivity which is based on their longevity and ability to work in harsh condition, and immobilization of enzyme is a useful method to achieve this goal [1]. Several methods and different kinds of supports have been used for immobilization, providing physical strength, stability, and enhancement of specificity/activity of enzymes [2–4]. Micro- and nanostructured silica and silicon are promising supports that offer the properties needed for not only biocatalysts [5, 6] but also nanovehicle-based drug delivery [7–9], tissue engineering [10, 11], and biosensors [12–14].

Natural silica with defined morphologies can be synthesized under mild conditions, without using extreme conditions, e.g., at elevated temperatures, high pressures, and/or strongly acidic or alkaline media [15]. Silica production from industrial process scales only upto megatons whereas from natural sources such as plant and other biological organism equals to gigatons. The process of precipitation and polymerization aided silica into the plant body with the formation of intra- as well as extracellular silica bodies [16–18]. Plants containing silica are identified as biosilicifiers and are classified as either Si accumulators (rice [*Oryza sativa*], horsetails [*Equisetum arvense*], sugarcane [*Saccharum officinarum L*.], etc.) or Si non-accumulators (less than 3 mg Si/g dry matter), such as most dicotyledons, including legumes [19, 20]. Study of incorporation of silica within the plant cell wall has been well documented by botanists and materials scientists.

Biocatalysts have found various applications in different areas such as environmental monitoring, biotransformation, diagnostics, pharmaceutical and food industries for their higher efficiency, continuous operations, and easy downstream processing [21–26]. The progress in material sciences presents the researchers to select the most appropriate carriers in terms of loading capacity, stability, and operational performance of the biocatalyst [25, 27]. Lignin peroxidase and horseradish peroxidase (HRP) immobilized on activated silica have been used for the removal of chlorolignins from Kraft paper effluent. α-Amylase was found to have high chemical and mechanical stability when immobilized onto nanostructures of high surface area and ordered arrangement [26, 28]. Peroxidases obtained from *ligninolytic fungi* have been shown to detoxify pesticide such as atrazine, dichlorophenol, and bromoxynil to less toxic compounds and can be applicable for various environmental processes [29]. Peroxidase produced by *Streptomyces thermoviolaceus* acts as a delignifying agent in the paper pulp industry, and also extracellular peroxidase from *Streptomyces avermitilis* removes the intense color from paper-mill effluent.

HRP enzyme has found application in several diagnostic applications in pharmaceutics and medicine, such as the detection of human immunodeficiency virus and cystic fibrosis [30]. Due to the inherent drawback of peroxidase enzyme of getting deactivated in the presence of hydrogen peroxide (its own essential substrate required by the enzyme to carry out its reaction), their application as biocatalysts in industrial processes is still limited [31]. In this study, we investigated covalent immobilization and co-immobilization chemistries with both derivatized porous silicon and biogenic silica microparticles in order to improve the oxidative stability of peroxidase. We co-immobilized the peroxidase along with cytochrome c onto the porous supports to improve its stability against H_2_O_2._ Also, the thermal stability of the immobilized and co-immobilized biocatalyst has been studied.

## Methods

### Chemicals

A commercial peroxidase, Baylase® RP, was kindly donated by Bayer Mexico (Puebla, Mexico). Crystalline silicon was a product from Cemat Silicon (Warsaw, Poland). 10-undecenoic acid, N-ethyl-N′-(3-dimethylaminopropyl) carbodiimide (EDC), N-hydroxysuccinimide (NHS), 2, 2′-azino-bis (3 ethylbenzothiazoline-6-sulphonic acid) (ABTS), Guaiacol, and Remazol brilliant blue were purchased from Sigma-Aldrich (St. Louis, MO, USA). Bradford reagent was from Bio-Rad (Hercules, CA, USA). All other chemical reagents were of analytical grade and were used without further purification.

### Fabrication of Porous Silicon Microparticles

Microparticles were prepared by electrochemical etching, using an electrolyte composed of an aqueous 48 % hydrofluoric acid (HF) solution (Sigma-Aldrich) and ethanol (EtOH, Fluka). The etching process was carried out at room temperature from <100> oriented, heavily doped p-type Si wafers of resistivity 0.002–0.005 Ω-cm. The wafer was etched in a 7-cm^2^ etching cell in 3:1 HF/EtOH solution with a constant current density of 142 mA cm^−2^ for 180 s. The porous layer was then lifted off by electropolishing in a 1:29 (*v*/*v*) solution of 48 % HF and EtOH for 120 s at a current density of 200 mA cm^−2^. The resulting porous layers were ultra-sonicated (ultrasonic cleaner; Thermal Fisher Scientific) in ethanol for 2 h to form the microparticles. The obtained porous silicon microparticles (PSi) had an average particle size in the range of 50–150 μm as estimated from their scanning electron microscopic (SEM) images.

### Biogenic Porous Silica Microparticles

Commercially available biogenic porous silica (BSiO_2_) (Concretio silicea bambuseae or “Tabasheer”) was purchased from Bristol Botanicals Ltd, UK. It was rotor-milled in a Fritsch Pulverisette P14 mill to a fine white powder with a d50 of 16 μm.

### Characterization of Porous Materials

The morphology of the porous materials was analyzed using a high resolution scanning electron microscopy. In order to determine the elemental composition of the biocatalyst, the technique of energy dispersive X- ray (EDX) diffraction was used. For quantitative measurement, the spectra were recorded on the above microscope and X- ray analyzer with additional JEOL 100 CX quantitative EDX instrumentation.

To study the surface chemical composition changes of the materials, their Fourier transform infrared (FTIR) spectra were collected at a resolution of 2 cm^−1^ on a Cary 640/660 FTIR spectrometer attached with an attenuated total reflection (ATR) accessory (Agilent Technologies, Mexico, Federal District, Mexico). The spectra were recorded in the wave number range of 500–4000 cm^−1^, accumulating 64 scans for each at a resolution of 4 cm^−1^. All ATR-FTIR spectra were recorded at ambient temperature.

The nitrogen adsorption-desorption isotherms of the silica and porous silicon particles were recorded using a Belsorp Mini-II sorbtometer at 77 K. The pore size distributions of the samples were determined from their adsorption isotherms using the BJH method and the mean pore size was obtained from the pore size distribution using desorption data and the Barret–Joyner–Halenda (BJH) method. For the case of nitrogen, the cross-sectional area is taken as 16.2 A2/molecule. The specific area was calculated from the Brunauer–Emmett–Teller (BET) equation.

### Enzyme Immobilization

Immobilization of MPs was done using the method proposed by Zhu et al. [32]. MPs were first subjected to heat treatment with 10-undecenoic acid in a micro-oven at 5 W for 4 min. The derivatized microparticles were rinsed consecutively with copious amounts of ethanol, dried and then analyzed. For immobilization, the microparticles were reacted with a mixture of freshly prepared 5 mM NHS and 50 mM EDC in phosphate buffer pH 6.0 along with peroxidase for 4 h. While for co-immobilization after undecenoic step, first cytochrome c (pH 6.8) was incubated with microparticles using EDC and NHS for an hour. Washed with phosphate buffer pH-6 to remove unbound cytochrome c. Cytochrome bound microparticles were then incubated with peroxidase using EDC and NHS for 4 h. All the immobilization was done at 4 °C in shaking condition. Finally, peroxidase-bound microparticles were washed with phosphate buffer three times for subsequent assays.

Enzyme immobilization on biogenic silica was carried out using adsorption technique by directly incubating the enzyme along with NHS/EDC following the same protocol thereafter as done with silicon microparticles.

### Determination of Enzyme Activity and Protein

Catalytic activity of peroxidase was determined by measuring the oxidation rate of ABTS at 25 °C in a 1 ml reaction mixture containing 60 mM phosphate buffer pH 5.0, 0.1 mM, ABTS and 1 mM H_2_O_2_. Reactions were initiated by adding H_2_O_2_ as the last component of the mixture. The initial rate of formation of the ABTS oxidation product was measured at 405 nm and converted to initial rate using ε = 36 mM^−1^cm^−1^. Kinetic absorbance measurements were performed with a UV–vis spectrophotometer model Camspec M105. The protein content was determined by Bradford method with the BioRad protein reagent.

### Stability of Peroxidase

Two different stabilities were tested for soluble, one-protein and two-protein preparations with porous silicon and biogenic microparticles. Thermal stability was measured by incubating the biocatalyst at 50 °C; while the oxidative stability was measured by incubating the biocatalyst in the presence of 1 mM H_2_O_2_. Residual activity was determined by taking aliquots of each sample at different time interval and assaying for enzymatic activity under the standard condition. The data were adjusted to a first-order rate model in order to calculate inactivation rate constants.

## Results and Discussion

### Nitrogen Adsorption Isotherm

**Fig. 1:**
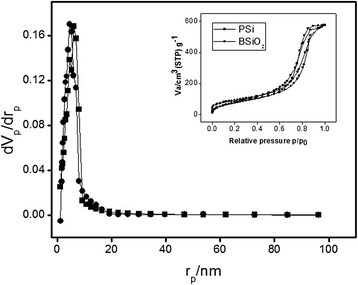
Pore size distribution of (*black square*) PSi and (*black circle*) BSiO_2_ microparticles and the *inset* shows the nitrogen adsorption desorption isotherms of these two mesoporous materials

The pore size distribution in the porous silicon and biogenic silica calculated from their N_2_ adsorption isotherms are presented as inset of Fig. 1. The N_2_ adsorption–desorption isotherms revealed for the porous silicon and biogenic silica are characteristic of monolayer–multilayer adsorption followed by capillary condensation at P/P_0_ = 0.99, that can be readily classified as type IV isotherms. The BSiO_2_ (•) presents a much narrower hysteresis loop centered on the desorption branch of the corresponding PSi (▪) microparticles. This is a partial confirmation of Cohan’s model based on the different shapes of the meniscus in adsorption and desorption [33]. The latter also indicates that both the samples are mesostructured materials. Moreover, the relatively sharp increase in volume adsorbed between P/P_0_ = 0.99 is an indication of the presence of uniform mesoporous as confirmed by the Barrett-Joyner-Halenda pore size distribution curves shown in Fig. 1. Key parameters for both the materials are provided in Table 1.

**Table 1:** N_2_ adsorption result for the porous materials. Surface area, pore volume, and average pore size of the microparticles

Sample	BET surface area (m^2^/g)	Total pore volume (cm^3^ g^−1^)	Average pore diameter (nm)
Porous silicon	287.11	0.889	12.38
Biogenic silica	323.85	0.8843	10.92

### Steps of Immobilization and Co-immobilization of Peroxidase

**Scheme 1:**
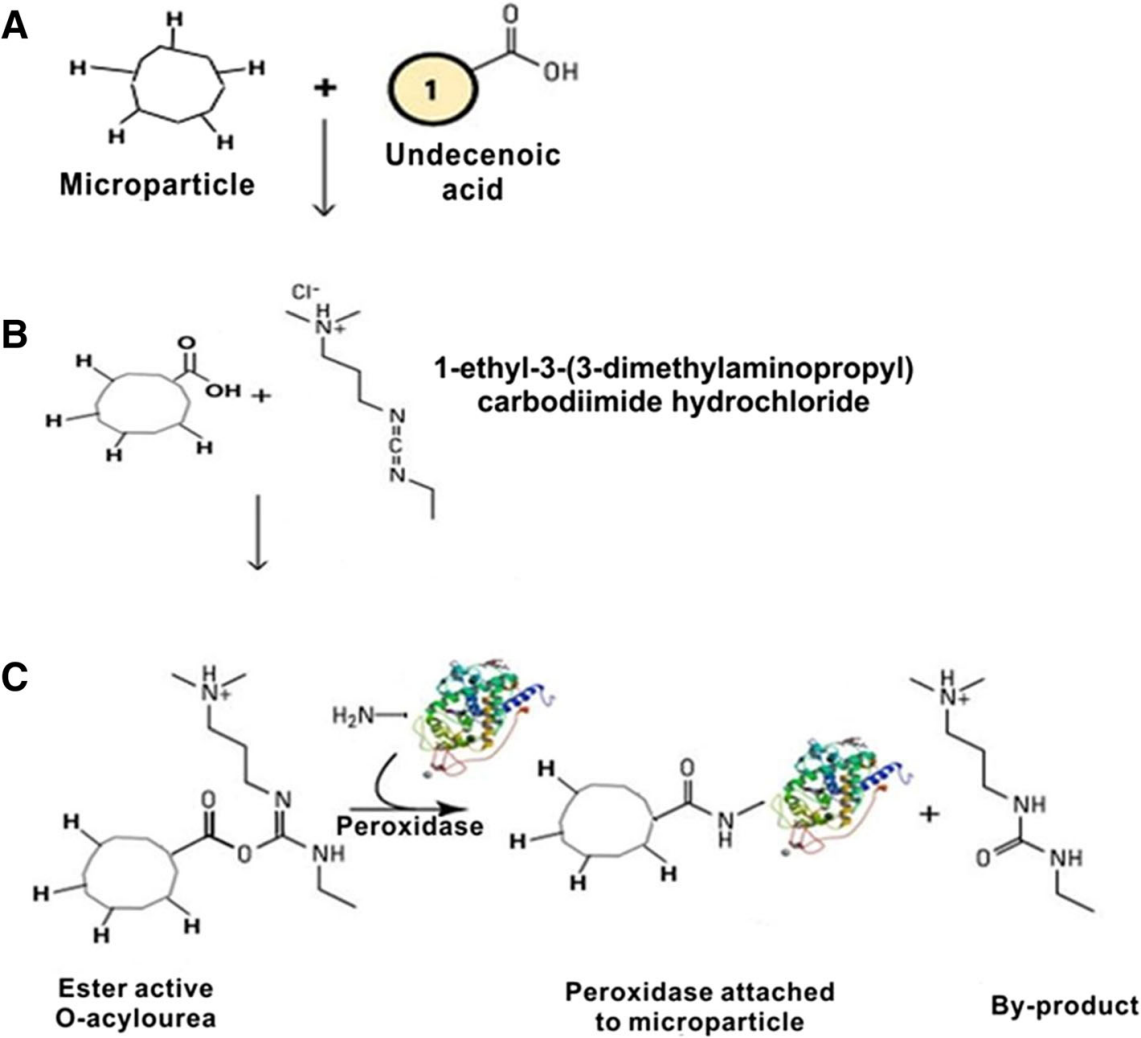
Microparticles were treated with undecenoic acid to get carboxy-terminated microparticles. Microparticles were then incubated with peroxidase along with 1-ethyl-3-(3-dimethylaminopropyl) carbodiimide (EDC) and N-hydroxysuccinimide (NHS)

**Scheme 2:**
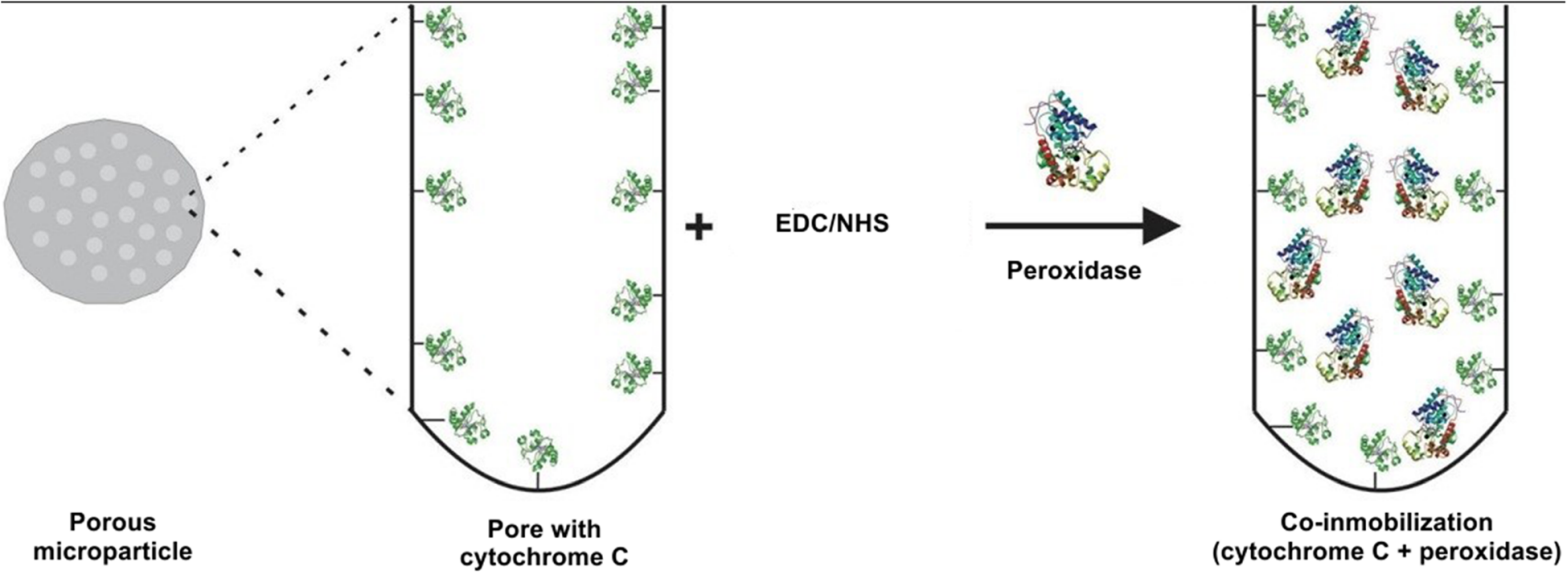
Microparticles treated with 10-undecenoic acid and then incubated with cytochrome c along with EDC and NHS. These cytochrome-immobilized particles were washed with phosphate buffer and then incubated with peroxidase, EDC, and NHS

Surface modification of support materials is commonly used for promoting the activity of immobilized enzymes. The chemical approach for attaching peroxidase to the microparticles surfaces is shown in Scheme 1. Carbodiimide chemistry is a popular method for crosslinking carboxylic acids and it works by activating carboxyl groups for direct reaction with primary amines via amide bond formation [34]. On PSi microparticles after monolayer formation with undecenoic acid, peroxidase was immobilized onto the microparticles by incubating in the presence of NHS and EDC. As shown in Scheme 2, similar steps were followed to first immobilize cytochrome c into the microparticles using carbodiimide chemistry and then to perform a second immobilization reaction with peroxidase. Whereas in case of BSiO_2_, the undecenoic acid step was omitted and immobilization/co-immobilizations was performed by straightforward incubation of the enzyme conjointly with NHS and EDC. The resulting biocatalysts are called one-protein and two-protein preparations, respectively.

### SEM and EDX Studies

**Fig. 2:**
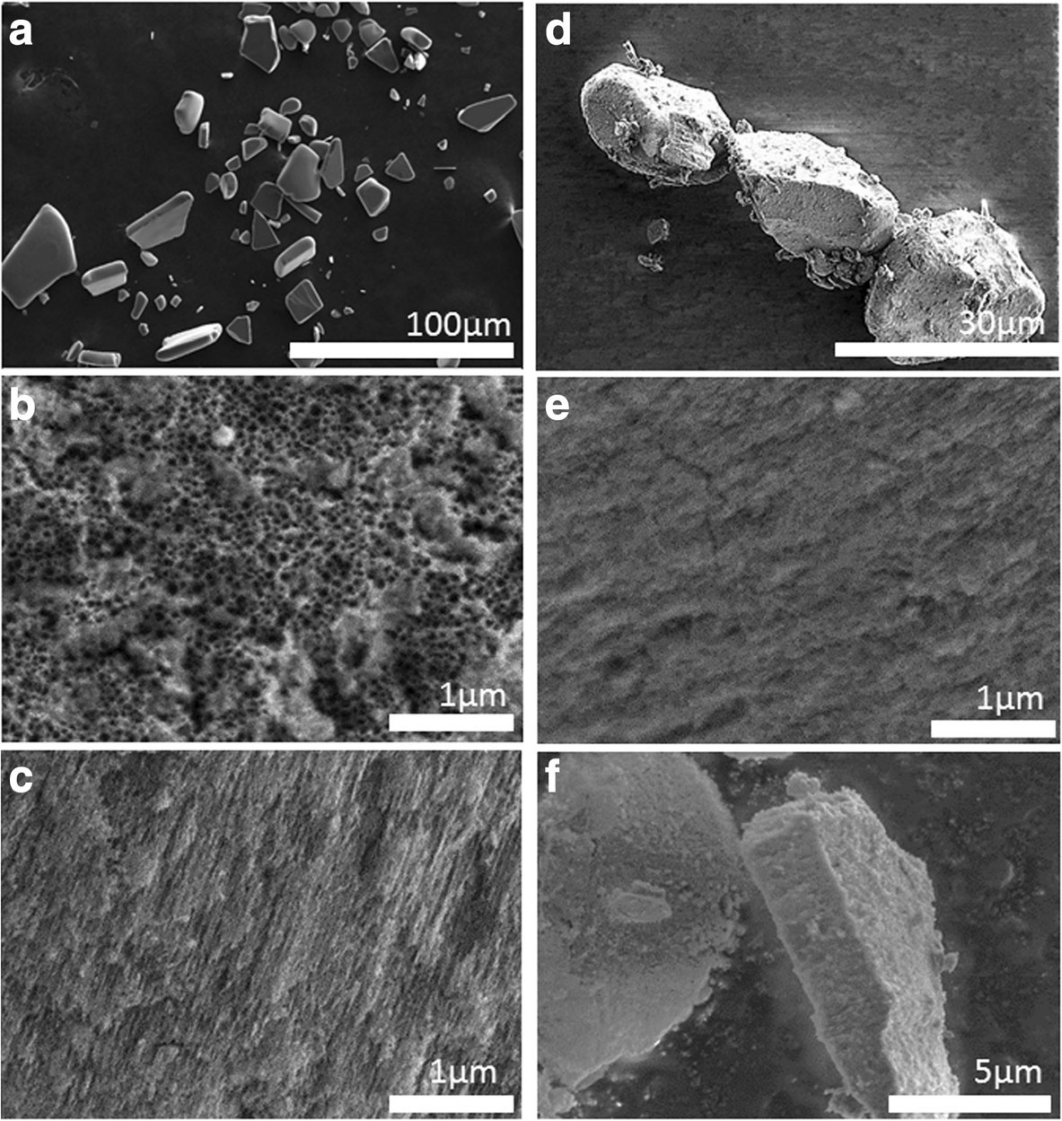
SEM image. (**a**, **d**) Top view. (**b**, **e**) Surface view. (**c**) 2f cross sectional of PSi microparticles and BSiO_2_ microparticles

**Fig. 3:**
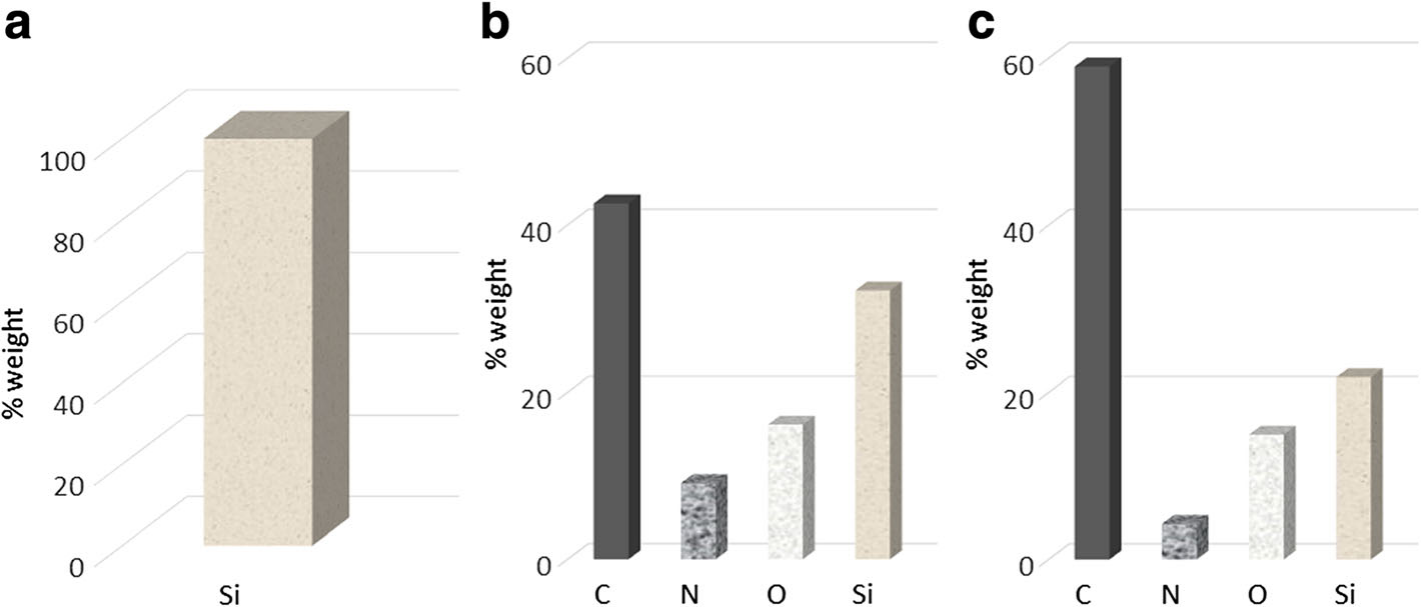
EDX results for PSi microparticles. **a** PSi microparticles, **b** cytochrome c immobilized to microparticles, and **c** peroxidase immobilized to microparticles

**Fig. 4:**
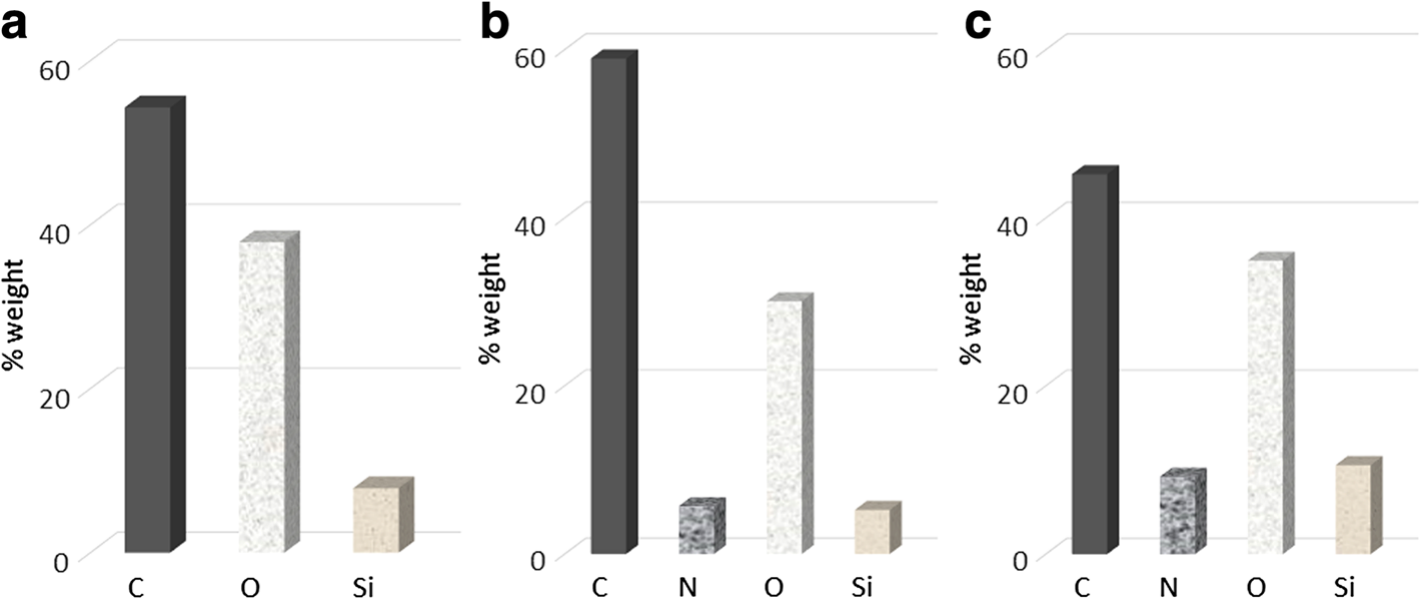
EDX results for BSiO_2_ microparticles. **a** BSiO_2_ microparticles, **b** cytochrome c immobilized to microparticles, and **c** peroxidase immobilized to microparticles

EDX coupled to SEM was used to characterize the biocatalysts. EDX provides information on elemental composition of the material surfaces; SEM gives the information regarding the size and morphology of the microparticles without complex sample preparation process. SEM analysis of the PSi and BSiO_2_ microparticles are presented in Fig. 2, Fig 2a) & 2d) shows the top view, 2b) & 2e) surface view and 2c) and 2f) show the magnified images of the pores of the corresponding microparticles. The sizes of the microparticles are in the range of 50–150 μm for PSi microparticles and 10–30 μm for biogenic silica sample. EDX spectra of different enzyme-containing PSi and biogenic silica microparticles are shown in Figs. 3 and 4, respectively. The appearance of C, N, and O peaks in the EDX spectrum of PSi after the immobilization process indicates that both proteins have been immobilized onto these microparticles. In the case of biogenic silica, the increase in C and O content as well as appearance of N peaks confirms the immobilization of the proteins. The most meaningful signal is the N peak which arises from the presence of protein in the material. The SEM coupled to EDX eventually provides a direct experimental evidence of the enzymes immobilization within the microparticles as well as particle size.

### FTIR Studies

**Fig. 5:**
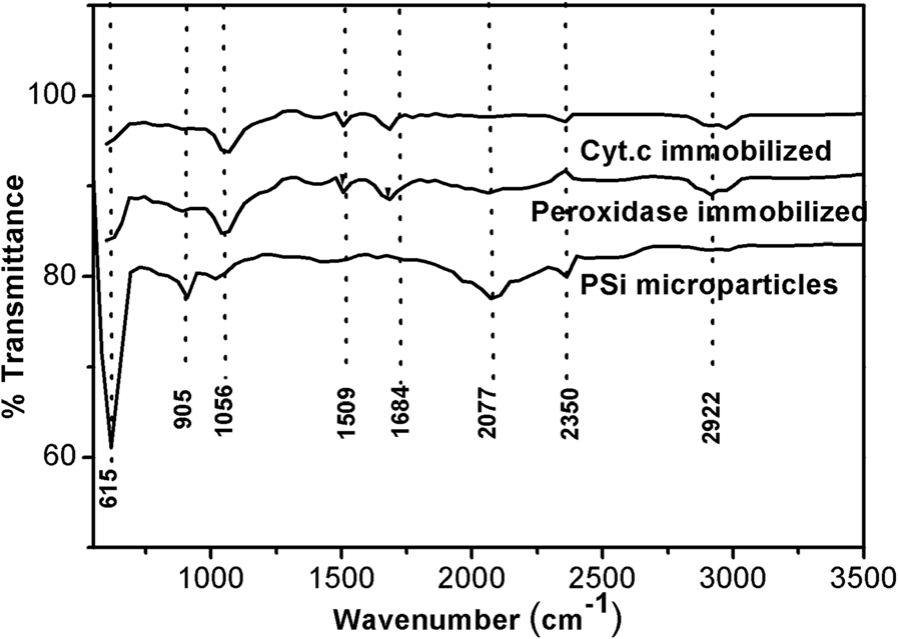
FTIR of one-protein and two-protein PSi-based biocatalysts

**Fig. 6:**
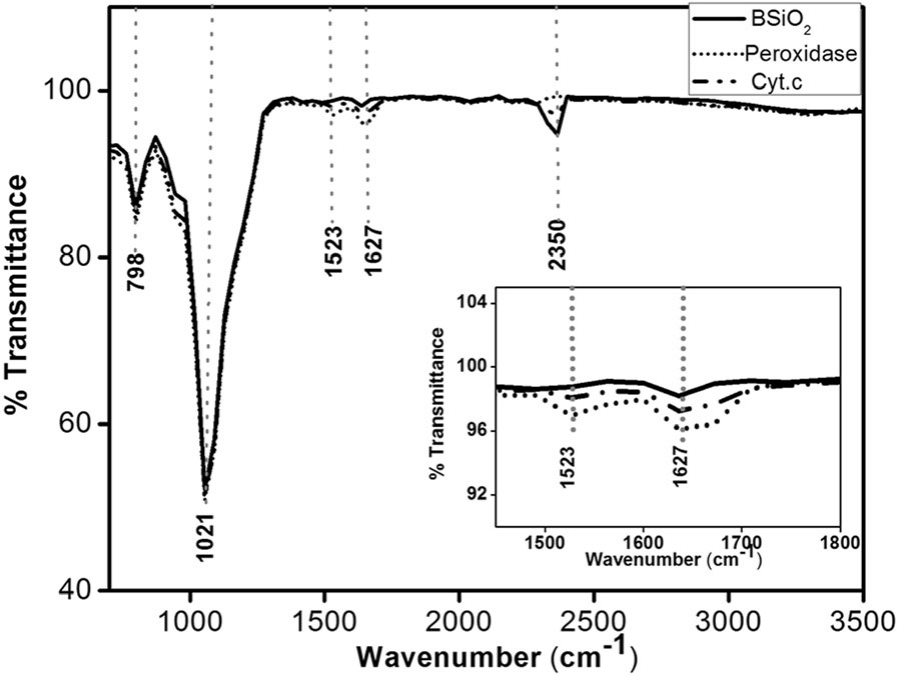
FTIR of one-protein and two-protein BSiO_2_-based biocatalysts

FTIR is a useful technique for investigating the surface-bound species and interface bonding of a chemically modified surface. Freshly prepared hydride-terminated PSi microparticles were functionalized with carboxylic acid-terminated monolayers by thermal hydrosilylation of 10-undecenoic acid. The FTIR absorbance spectrum shows the absorbance characteristic of the hydride-terminated surface of a (Si-H_2_) scissor mode at 910 cm^−1^ and a mode at 626 cm^−1^ assigned to Si-H_*x*_ vibrations. The presence of 1025 cm^−1^ band associated to symmetric stretching mode of Si-O demonstrates the formation of siloxane bonds on PSi internal surface [35]. The band revealing in between 1000 and 1200 cm^−1^ belongs to the Si-O-Si asymmetric stretching mode, typical for a siloxane network or chains. The band consists of several overlapping peaks that correspond to Si-O-Si in different configurations [36]. It is seen that in all the spectra shown in Figs. 5 and 6, there are peaks centered at about 2350 cm^−1^ which is a typical fingerprint of CO_2_. The unique peaks (marked by arrows) that appeared in one-protein and two-protein biocatalysts correspond to the amide I and amide II bands of the protein infrared spectrum. The amide I band (ranging from 1600 to 1700 cm^−1^) is mainly associated with the C–O stretching vibration (70–85 %) and is directly related to the backbone conformation. Amide II results from the N–H bending vibration (40–60 %) and from the C-N stretching vibration (18–40 %). The peaks corresponding to BSiO_2_ and the biocatalyst are shown in Fig. 5. A peak at 798 cm^−1^ could be assigned to the bending mode of a secondary amine (−NH). All other detectable peaks are the same as found in PSi biocatalyst sample. The prominent IR peaks revealed for the PSi and BSiO_2_ are listed in Tables 2 and 3, respectively.

**Table 2:** FTIR peaks for PSi microparticles. Position and identification of important FTIR bands of the PSi-based biocatalysts

PSi (cm^−1^)	+ cytochrome c (cm^−1^)	+peroxidase (cm^−1^)	Group	Vibrational mode	References
615	–	–	Si-H_*x*_	Vibrational mode	[35]
905	–	–	SiH_2_	Scissor	[35, 37]
1025	–	–	Si-O		[35, 37]
–	1056	1056	Si-O-Si	Asymmetric stretching	[35, 36, 38]
–	1509	1509	N–H, C-N	Bending, stretching	[38]
–	1684	1684	C = O	Stretching	[37, 38]
2350	2350	2350	Ambiental CO_2_	Asymmetric stretching	[39]
–	2922	2922	CH_2_		[37, 40, 41]

**Table 3:** FTIR peaks for BSiO_2_. Position and identification of important FTIR bands of the BSiO_2_-based biocatalysts

BSiO_2_ (cm^−1^)	+ cytochrome c (cm^−1^)	+ peroxidase (cm^−1^)	Group	Vibrational mode	References
580	580	580	–	–	–
798	798	798	-NH	Bending	[35]
1054	1054	1054	Si-O-Si	Asymmetric stretching	[35, 36]
–	1523	1523	N–H, C-N	Bending, stretching	[37]
–	1647	1647	C = O	Stretching	[36, 37]
–	2350	2350	Ambiental CO_2_	Asymmetric stretching	[38]

### Comparison of Peroxidase Activity in One-Protein and Two-Protein Biocatalysts

Protein loading and biocatalytic activity of the one-protein and two-protein biocatalysts for the two types of microparticles are presented in Table 4. Biogenic silica microparticles are able to immobilize a slightly higher amount of peroxidase compared to PSi microparticles. When the enzyme is co-immobilized with cytochrome c, the peroxidase load does not decrease, thus suggesting a bilayer formation. The activity found in the microparticles is 24–26 % of the expected activity according to enzyme load for the one-protein biocatalysts. However, when co-immobilizing with cytochrome c, the activity increases to 52–53 % of the expected activity. Cytochrome c is a small heme-protein found loosely associated with the inner membrane of the mitochondrion. It is capable of undergoing oxidation and reduction reaction. It is important to point out that cytochrome c is able to perform peroxidase-like reactions [42] but its activity is several order magnitude lower than peroxidases. The aim of this work was to induce cytochrome c to act as a reducing agent for the removal of oxidative equivalents, increasing the half-life of peroxidase and thus reflecting on the higher activity found for the two-protein biocatalysts.

**Table 4:** Comparative study of peroxidase activity in one-protein and two-protein biocatalysts. Table showing the payload and activity of the one protein and two proteins immobilized onto PSi and BSiO_2_ microparticles

Samples	Expected activity in support (U/mg)	Activity found in support (U/mg)	Protein (peroxidase) (μg/mg)	Protein (cytochrome c) (μg/mg)
PSi (one protein)	48.26	12.43	53	–
PSi (two proteins)	57.7	30.97	128	53
BSiO_2_ (one protein)	53	12.98	57	–
BSiO_2_ (two proteins)	34	17.9	72	56

### Thermal Stability of Peroxidase

**Fig. 7:**
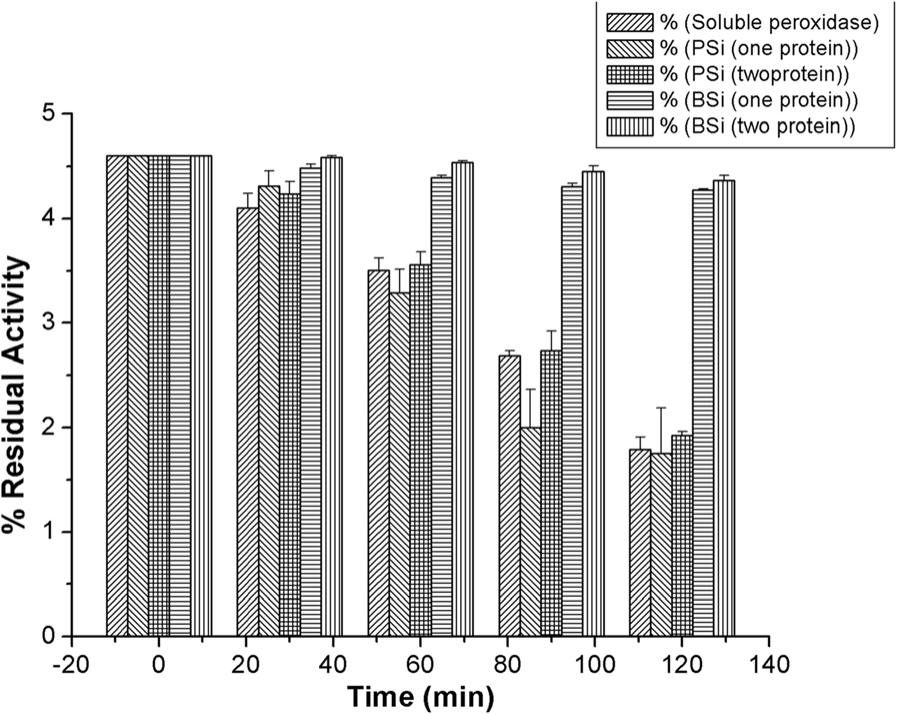
Residual activity of soluble peroxidase and one-protein and two-protein biocatalysts during incubation at 50 °C

Effect of temperature on the activity of free enzyme as well as one-protein and two-protein biocatalysts was investigated. Reactions were carried out at pH 6.0 and temperature influence was studied at 50 °C for a time interval of 0–4 h (Fig. 7). The first-order inactivation rate constants (*k*_inact_) are presented in Table 5. One-protein and two-protein PSi microparticles display the same thermal stability as the soluble enzyme. This lack of thermal stabilization could be attributed to the microenvironment within the PSi microparticles containing residual surface hydride groups, as confirmed from the FTIR studies even after the immobilization of peroxidase enzyme [43]. On the other hand, both one-protein and two-proteins BSiO_2_ biocatalysts showed an increased thermal stability, with inactivation rate constants 23-fold smaller than that of soluble enzyme. The above results point out that the biogenic silica matrix preserved the structure of the enzyme, protecting the enzyme from conformational changes caused by heating. Co-immobilization does not seem to exert a protective effect against thermal denaturation of the peroxidase in either biogenic silica or PSi microparticles.

**Table 5:** Thermal deactivation rate constants. First-order inactivation rate constants of soluble peroxidase and one-protein and two-protein biocatalysts during incubation at 50 °C

Sample	*k*_inact_ (h^−1^)	*R* ^2^
Soluble	0.023	0.9848
PSi(I)	0.026	0.9504
PSi(Co)	0.023	0.9815
BSiO_2_(I)	0.001	0.9649
BSiO_2_(Co)	0.001	0.9414

### Stability of Peroxidase in the Presence of Hydrogen Peroxide

**Fig. 8:**
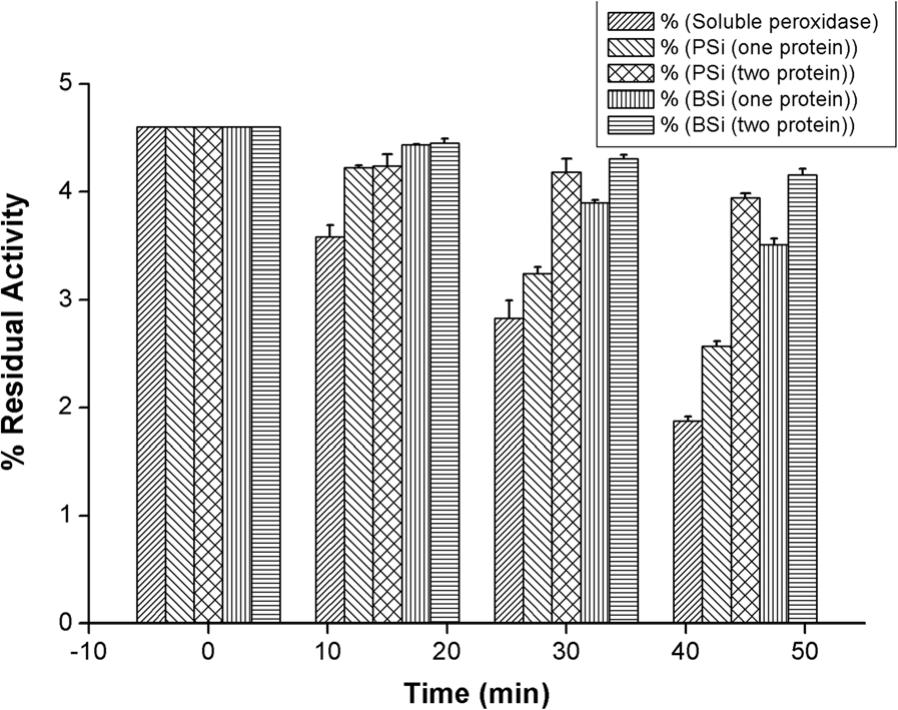
Residual activity of one-protein and two-protein biocatalysts upon incubation with 1 mM H_2_O_2_

Peroxidases gets inactivated during catalytic turnover or in the absence of reducing substrates [44]. The stability of soluble peroxidase was determined by incubating the protein with a catalytic concentration of hydrogen peroxide (1 mM H_2_O_2_) for a period of time (Fig. 8). The time course of oxidative inactivation was followed by measuring the residual activity of the peroxidase with ABTS as substrate. After 45 min, the soluble peroxidase lost 85 % of its activity. In the case of the microparticle-based biocatalysts, the one-protein and two-protein PSi biocatalyst retained 13 and 51.8 % activity, respectively. Similarly, the one-protein and two-protein BSiO_2_ biocatalysts retained 33.4 and 63.9 % activity after 45 min of incubation. The first-order inactivation rate constants (*k*_inact_) are presented in Table 6. The differences in inactivation rate constant observed between one-protein and two-proteins biocatalysts imply that co-immobilization of cytochrome c significantly enhances the peroxidase stability in comparison to the one-protein biocatalyst; and the improvement was more significant with the biogenic silica, thus reinforcing the notion that BSiO_2_ is a better support for peroxidase immobilization. The strategy of co-immobilizing cytochrome c and peroxidase was aimed to improve the stability of peroxidase in presence of H_2_O_2_ which is the enzyme-activating substrate and is also involved in a mechanism-based process described as suicide inactivation. From the above results, it can be calculated that the two-protein biocatalyst are between four and six times more stable towards oxidative inactivation than the soluble enzyme.

**Table 6:** Inactivation rate constants of peroxidase in the presence of H_2_O_2._ First-order inactivation rate constants of soluble peroxidase and one-protein and two-protein biocatalysts during incubation with 1 mM H_2_O_2_

Sample	*k*_inact_ (h^−1^)	*R* ^2^
Soluble	0.056	0.9949
PSi(I)	0.047	0.9756
PSi(Co)	0.014	0.9284
BSiO_2_(I)	0.025	0.9824
BSiO_2_(Co)	0.009	0.9858

## Conclusions

In this work, we presented the covalent co-immobilization of a commercial peroxidase and a redox-active protein onto porous silicon and biogenic silica microparticles, for enhancing the operational properties of the resulting biocatalyst. By directly comparing silicon and silica structures with similar surface areas, pore volumes, and pore size distributions, the effects of differing chemical micro-environments could be explored. The participation of cytochrome c as a reducing agent for the removal of active oxygen in two protein biocatalyst leads to a significant decrease in self-inactivation characteristic of the peroxidase. Although co-immobilization fails to demonstrate a significant role in improving the thermal stability of the immobilized peroxidase, the biogenic silica exhibits a relatively higher protective effect as compared to porous silicon microparticles. Obtained results indicate that co-immobilization strategy and the use of biogenic silica material as support enhance the functional behavior of the peroxidase and give insight into the strategy for improving the oxidative stability of other peroxidases of biotechnological interest.

## Abbreviations

ABTS: 2, 2′-azino-bis (3 ethylbenzothiazoline-6-sulphonic acid)
ATR: attenuated total reflection
BET: Brunauer–Emmett–Teller
BSiO_2_: biogenic silica
BSiO_2_(Co): cytochrome c and peroxidase immobilized onto biogenic silica microparticles
BSiO_2_(I): peroxidase immobilized onto biogenic silica microparticles
Cyt c: cytochrome c
EDC: N-ethyl-N′-(3-dimethylaminopropyl) carbodiimide
EDX: energy-dispersive X-ray spectroscopy
EtOH: ethanol
FTIR: Fourier transform infrared
H_2_O_2_: hydrogen peroxide
HF: hydrofluoric acid
HRP: horseradish peroxidase
NHS: N-hydroxysuccinimide
PS: porous silicon
PSi(Co): cytochrome c and peroxidase immobilized onto porous silicon microparticles
PSi(I): peroxidase immobilized onto porous silicon microparticles
SEM: scanning electron microscopy
UV–vis: ultraviolet visible
